# Correction: The influence of model building schemes and molecular dynamics sampling on QM-cluster models: the chorismate mutase case study

**DOI:** 10.1039/d6cp90040b

**Published:** 2026-03-12

**Authors:** Donatus A. Agbaglo, Thomas J. Summers, Qianyi Cheng, Nathan J. DeYonker

**Affiliations:** a Department of Chemistry, University of Memphis Memphis TN 38152 USA ndyonker@memphis.edu

## Abstract

Correction for ‘The influence of model building schemes and molecular dynamics sampling on QM-cluster models: the chorismate mutase case study’ by Donatus A. Agbaglo *et al.*, *Phys. Chem. Chem. Phys.*, 2024, **26**, 12467–12482, https://doi.org/10.1039/D3CP06100K.

The authors would like to amend the results in the subsection “Schemes for selection of frames for the QM-cluster models from MD trajectories”. For Schemes 2–7 (S_2_–S_7_), it was originally written that the RMSD value was computed with respect to the X-ray crystal structure. However, in all cases, the RMSD value was computed with respect to the initial equilibrated structure from the molecular dynamics simulation.

After processing the 250 QM-cluster models to prepare some new data analysis for a forthcoming manuscript, we found some incorrect values in our data.

In the reported dataset:

Scheme 1, frame 20 000:

Old Δ*G*^‡^ = 9.60 kcal/mol, corrected Δ*G*^‡^ = 12.52 kcal mol^−1^

Old Δ*G*_rxn_ = −15.14 kcal mol^−1^, corrected Δ*G*_rxn_ = −10.33 kcal mol^−1^

Scheme 4, frame 4591:

Old Δ*G*^‡^ = 11.06 kcal mol^−1^, corrected Δ*G*^‡^ = 11.20 kcal mol^−1^

Old Δ*G*_rxn_ = −19.92 kcal mol^−1^, corrected Δ*G*_rxn_ = −12.02 kcal mol^−1^

Scheme 6, frame 4114 had subtle issues with the transition state search that sneaked into the publication. We have redone the computations and obtained the results reported here.

Old Δ*G*^‡^ = 19.07 kcal mol^−1^, corrected Δ*G*^‡^ = 10.62 kcal mol^−1^

Old Δ*G*_rxn_ = −19.42 kcal mol^−1^, corrected Δ*G*_rxn_ = −17.08 kcal mol^−1^

Scheme 8 frame 15 461:

Values of Δ*G*^‡^ = 9.05 kcal mol^−1^ and Δ*G*_rxn_ = −15.99 kcal mol^−1^ are correctly labeled in Table S9, but incorrect values were used in Tables 1 and 2. This has now been fixed.

New versions of Tables 1 and 2 are provided below.

A few of the selected 30 frames from S_2_, S_3_, and S_4_ did not meet the criteria for each scheme and were incorrectly included in those schemes: Frames 101, 2322, 15 246, and 17 817 in S_2_; frames 758, 4165, 6412, 12 729, and 18 630 in S_3_; frames 232, 806, 8886, 9662, and 18 831 in S_4_. We have removed the 14 incorrect frames from their relevant schemes and moved them into a new Scheme 9 (S_9_) that is essentially another route for random frame selection. As expected, S_9_ kinetics and thermodynamics are qualitatively similar to S_1_, where frames were selected at a constant interval of 1000 MD steps. Their inclusion into the superset of randomly selected frames in Table 2 (now labeled S_1_ + S_2_ + S_7_ + S_9_) has almost no effect on the final results.

Some of the frames selected in the *k*-means clustering of Scheme S_5_ were also misclassified. Of the 30 selected frames, 10 belonged to the first cluster (C_1_) where frames mostly had the largest RMSD of all heavy atoms compared to the initial MD structure, 14 belonged to C_2_, and only 6 belonged to C_3_, the cluster where frames generally had the smallest RMSD compared to the MD structure.

Changes to the final statistical metrics are not significant and the conclusions of the published work remain valid. Interestingly though, a new trend in Table 1 seems to emerge where activation energies increase with respect to increasing cluster number in Scheme S_5_. However, this relationship disappears when the QM-cluster models of a larger number of frames are partitioned into the three *k*-means clusters used for S_5_ (Table 2).

**Table 1:** Mean free energies of activation and reaction for the various MD frame selection schemes. *k*-means clusters are labelled with a C. (All values in kcal mol^−1^)

Scheme	Cluster	# of frames	Mean Δ*G*^‡^	*σ*	Mean Δ*G*_rxn_	*σ*
S_1_		20	10.22	2.92	−15.99	4.10
S_2_		26	10.03	2.20	−16.36	3.41
S_3_		25	9.93	2.80	−15.10	3.18
S_4_		25	10.22	1.96	−15.62	2.84
S_5_		30	10.29	3.05	−15.57	3.09
	C_1_	10	9.90	2.74	−16.78	3.12
	C_2_	14	10.37	3.65	−15.25	3.21
	C_3_	6	10.76	1.51	−14.31	1.83
S_6_		40	10.02	1.91	−15.18	2.98
S_7_		40	10.74	2.69	−15.35	3.52
S_8_		30	10.96	2.69	−13.82	3.36
	C_1_	10	10.83	1.95	−13.85	2.30
	C_2_	10	10.80	3.94	−13.57	4.15
	C_3_	10	11.25	1.53	−14.06	3.34
S_9_		14	10.13	2.65	−15.33	3.84
Full set		250	10.31	2.57	−15.32	3.40

**Table 2:** Mean free energies of activation and reaction for the expanded schemes. The individual *k*-means clusters are labelled XC. (All values in kcal mol^−1^)

Scheme	Cluster	# of frames	Mean Δ*G*^‡^	*σ*	Mean Δ*G*_rxn_	*σ*
S_1_ + S_6_ + S_7_ + S_9_		114	10.32	2.51	−15.40	3.51
XS_2_		148	10.13	2.66	−15.54	3.45
XS_3_		176	10.34	2.60	−15.38	3.51
XS_4_		186	10.41	2.64	−15.29	3.38
XS_5_						
	XC_1_	92	10.28	2.64	−16.10	3.96
	XC_2_	89	10.20	2.70	−14.88	3.00
	XC_3_	69	10.50	2.28	−14.85	2.83
XS_8_						
	XC_1_	77	10.46	2.53	−15.32	3.59
	XC_2_	81	10.17	2.69	−14.92	3.33
	XC_3_	92	10.32	2.49	−15.69	3.24

A few of the output structures in the Electronic supplementary information document are also missing. An updated version of the SI (also incorporating the aforementioned corrections to frames 4114, 4591, and 20 000) with all optimized PDB structures is provided at https://github.com/natedey/cm-MD_to_QM.

The Royal Society of Chemistry apologises for these errors and any consequent inconvenience to authors and readers.

